# p53 protein as a prognostic indicator in breast carcinoma: a comparison of four antibodies for immunohistochemistry.

**DOI:** 10.1038/bjc.1996.6

**Published:** 1996-01

**Authors:** G. M. Horne, J. J. Anderson, D. G. Tiniakos, G. G. McIntosh, M. D. Thomas, B. Angus, J. A. Henry, T. W. Lennard, C. H. Horne

**Affiliations:** Department of Pathology, Royal Victoria Infirmary, University of Newcastle upon Tyne, UK.

## Abstract

**Images:**


British Journal of Cancer (1996) 73, 29-35
© 1996 Stockton Press All rights reserved 0007-0920/96 $12.00

### p53 protein as a prognostic indicator in breast carcinoma: a comparison of four antibodies for immunohistochemistry

GM Horne[1], JJ Anderson[1], DG Tiniakos[1], GG McIntosh[1], MD Thomas[1], B Angus[1], JA Henry[1], TWJ Lennard[2] and CHW Horne[1]

*Departments of [1]Pathology and [2]Clinical Surgery, Royal Victoria Infirmary, University of Newcastle upon Tyne NE1 4LP, UK.*

**Summary** We examined the reactivity of four p53-specific monoclonal antibodies - PAb 1801, p53-BP-12, DO7 and CM1 - on sections of formalin-fixed tissue collected from 245 breast carcinomas. Immunodetection of p53 varied between 37.6% and 46.6%. The greatest variation was observed among lobular carcinomas and low-grade tumours in which immunodetection varied between 8.3% and 27.3%. In contrast, immunodetection of p53 in invasive ductal carcinomas was subject to a lower degree of variability with between 40.6% and 49.7% of these tumours proving to be positive. In general, we found antibodies PAb 1801 and DO7 to be the most effective in immunolocalising p53. Immunodetection of p53 with each of the four antibodies was found to correlate strongly with tumour grade. In survival analysis, the results gained using antibody PAb 1801 proved to be of greatest statistical significance and to provide the strongest index of prognosis. A significant relationship was observed between immunodetection of p53 with each of the four antibodies and poor responsiveness to endocrine therapy. In addition, relationships were also observed between p53 immunostaining and tumour oestrogen receptor (ER) status as well as c-*jun* expression. We observed no correlation between abnormalities of the p53 and the Rb gene products or between elevated c-*erb*B-2 or epidermal growth factor receptor (EGFR) expression and immunodetection of p53.

**Keywords:** p53; tumour-suppressor gene; immunohistochemistry; breast carcinoma

Prediction of likely outcome in breast cancer has acquired greater importance following the demonstration by meta-analysis that chemotherapy provides enhanced 10 year survival rates (Early Breast Cancer Trials Collaborative Group, 1992). Accurate prediction of outcome would allow rational allocation of patients to appropriate treatment regimens. Lymph node status is accepted as a primary prognostic factor but it is recognised that greater predictive power would be desirable, and numerous potential prognostic factors have been examined, for example tumour grade, hormone receptor status and c-*erb*-B2 protein expression. (Barnes *et al*., 1989; Neville *et al*., 1990; Galea *et al*., 1992). Cattoretti *et al*. (1988) were the first to demonstrate that p53 gene expression might serve as a prognostic indicator in breast cancer. This has subsequently been confirmed by other workers who have shown that p53 protein overexpression determined by immunohistochemistry gives significant supplementary information regarding likely outcome (Iwaya *et al*., 1991; Sawan *et al*., 1992; Barnes *et al*., 1993; Yamashita *et al*., 1993).

The p53 gene is located on the short arm of chromosome 17 and it encodes a 53 kDa nuclear phosphoprotein involved in the control of cell proliferation (Baker *et al*., 1989). The exact function of the p53 protein is not fully understood but it may play a role in DNA replication and regulation of transcription (Levine *et al*., 1991). Recent studies have shown that p53 in its 'wild' form may act as a tumour-suppressor gene (Lane and Benchimol, 1990). The precise mechanism of p53 tumour suppression is not known. Recent evidence suggests that p53 indirectly inhibits cell proliferation by regulating the transcription of other unidentified genes (Kern *et al*., 1992). The p53 gene has been found mutated and/or deleted in a wide range of tumours (Mulligan *et al*., 1990; Levine *et al*., 1991). Functional inactivation of the p53 gene by mutation or allelic loss appears to be one of the commonest genetic abnormalities in human cancer (Lane, 1992). Structural abnormalities of the p53 gene have been detected in sarcomas, leukaemias, colonic, lung, oesophageal and liver carcinomas (Vogelstein and Kinzler, 1992). Mutation leads to altered protein conformation and increased half-life of the p53 gene product, resulting in detectability by immunohistochemistry. Recent immunohistochemical studies have shown that the detection rate of mutated p53 protein in breast cancer ranges between 13% and 53.5% (Cattoretti *et al*., 1988; Sawan *et al*., 1992; Martinazzi *et al*., 1993; Yamashita *et al*., 1993).

The aim of the present study was to assess the expression of the p53 gene in a large series of routinely processed human breast carcinomas and its relation to tumour variables, prognostic factors and patient outcome. In doing so we compared the effectiveness of four different antibodies commonly used in immunolocalisation studies of p53.

### Materials and methods

### *Tissues*

Our study included 245 resected breast carcinomas from 245 female patients (mean age: 57.02 years with a standard deviation of 13.87, range: 24-98 years) attending the Royal Victoria Infirmary, Newcastle upon Tyne, UK between 1983 and 1990. Tumour tissues were received fresh in the Department of Pathology, were dissected and block-sized pieces were fixed overnight in neutral buffered formalin. Following primary fixation the blocks were further trimmed and post-fixed in formal sublimate (saturated aqueous mercuric chloride and 37% formaldehyde, 9:1) for approximately 3 h. Blocks were then routinely paraffin embedded. The histological classification of the tumours was based on the WHO criteria and the grading was according to the criteria of Bloom and Richardson modified by Elston (Elston and Ellis, 1991). The histological diagnosis, tumour size and lymph node status was determined during routine pathological assessment. Tumour grade was determined by one observer.

Patients were followed up for a maximum of 103 months. The mean follow-up time was 39 months. Patient follow-up data were acquired by reference to the case notes. Relapse-free and overall (time to death) survival were measured from the date of surgery. Only deaths due to breast cancer were

Correspondence: JJ Anderson
Received 27 January 1995; revised 17 July 1995; accepted 1 August 1995

considered for the purposes of the study. Patients dying of causes other than breast cancer were censored at the end of their observation times. Relapse was considered as any evidence of metastasis or local recurrence.

The patient group has been the subject of other prognostic studies. Data concerning oestrogen receptor (ER), progesterone receptor (PgR) and epidermal growth factor receptor (EGFR) status determined by frozen section immunohistochemistry (Elston and Ellis. 1991; Piggott *et al.*, 1991; Sawan *et al.*, 1992), expression of c-erbB-2 and c-jun oncoproteins and expression of Rb protein determined by paraffin section immunochemistry were available (Corbett *et al.*, 1990; Anderson *et al.*, 1994; Tiniakos *et al.*, 1994). In addition, a small subgroup of patients ($n = 17$) has been previously assessed regarding response to endocrine therapy on relapse, and these data were also available for comparative studies. The minimum criterion for response to hormonal therapy on relapse was disease that did not progress for at least 6 months (Henry *et al.*, 1991).

### *Immunohistochemical method and antibodies*

The streptavidin–biotin complex (sABC) (Dakopatts, Denmark) immunoperoxidase techique was used on 5-$\mu$m-thick paraffin sections. Table 1 displays in detail the four antibodies used for the detection of p53 protein. The antibodies used were selected on the basis of reactivity with paraffin-embedded tissue and at the time were the only reagents effective for this application. The ideal immunostaining conditions for each of the antibodies had been established in preliminary experiments. The microwave antigen retrieval technique preceded immunohistochemical staining using DO7 and PAb 1801 monoclonal anti-p53 antibodies (Shi *et al.*, 1991). Briefly, the tissue sections were placed in sodium citrate solution (0.01 M, pH 6.0) and then incubated twice for 5 min in an 800 W microwave oven. The chromogen of choice was diaminobenzidine (DAB).

Positive controls consisted of breast cancer sections with known detectable p53 protein. In each case, a section in which the primary antibody incubation was omitted was used as a negative control.

### *Scoring*

The cellular immunoreaction was scored on the basis of the intensity of the specific nuclear staining on a five-point scale: 0, no staining; 1, equivocal or very weak staining; 2, definite staining of moderate intensity; 3, strong staining; 4, very strong staining. Only tumours scoring 2 or more were regarded as 'positive'. The proportion of cells staining was not taken into account. Some tumours showed occasional isolated individual cells with strong nuclear staining. These were regarded as probable apoptotic cells and discounted. The immunoreaction was assessed independently by two histopathologists.

### *Statistical analysis*

The chi-squared test, together when appropriate with Yeat's correction, was used for the correlations between the different tumour and patient variables. Log-rank tests were used for survivial analyses. All analyses were carried out using Solo Statistical Software (BMDP Statistical Software, USA).

### Results

The apparent rate of p53 protein overexpression/stabilisation in breast carcinomas varied from 37.6% to 46.6% depending on the anti-p53 antibody used (Table II, Figure 1). The highest proportion of cases showing nuclear p53 immunostaining was detected using the antibody PAb 1801, while the lowest number of p53-positive cases was observed using the polyclonal antibody CM1. The nuclear immunostaining produced using CM1 was often accompanied by the presence of cytoplasmic reactivity and non-specific background staining. In addition, in several instances using CM1, cytoplasmic reactivity was observed in the absence of nuclear staining despite the fact that positive nuclear reactivity was produced by each of the other antibodies. In subgroups stratified by histological type, the rate of p53 protein immunodetection varied from 40.6% to 49.7% in invasive ductal carcinomas (IDCs) ($n = 205$) and 8.3% to 27.3% in invasive lobular carcinomas (classical type) (ILCs) ($n = 15$). Colloid carcinomas ($n = 7$), with the exception of one case, were p53 negative with all the antibodies used. One out of three medullary carcinomas and one out of five *in situ* ductal carcinomas showed positive p53 immunoreactivity, as detected with all four antibodies. One tubular and one squamous carcinoma included in the study were p53 negative.

As expected a very strong and highly significant correlation for positive staining with any of the four p53 antibodies was observed with each of the others. However, in each cross-tabulation analysis a number of cases with discrepant staining results were observed i.e. scored p53 positive with one antibody and negative with another, or vice versa. The strongest correlation of scoring results was observed for DO7 compared with PAb 1801; by contrast when CM1 and p53-BP-12 were compared, 23% of cases were discordant. Fifty-seven out of 245 cases (23.2%) were concordant for positive staining for all four antibodies, while 89/245 (36.3%) were negative for all four; this leaves a pool of 41.5% cases in which some degree of discordancy is observed.

Lymph node status was the most powerful predictive factor for both time to relapse and time to death ($P = 0.021$ and $P = 0.0001$ respectively). Using the monoclonal antibody PAb 1801, p53 expression was associated with poorer overall survival when all patients were considered ($P = 0.017$); in subgroups stratified by node status this observation was sustained only in the node-negative group ($P = 0.037$) (Figure 2). When relapse-free survival was assessed, immunostaining for p53 using PAb 1801 was not associated with poorer prognosis. Immunostaining for p53 protein did not relate significantly to survival or relapse-free survival, using DO7, p53-BP-12 and CM1 antibodies. When groups of patients were defined according to positivity for any antibody, compared with negativity for all antibodies, no survival differences for either of these groups was observed, either taking the data as a whole, or by stratification according to node status.

Using each one of the four antibodies, p53 expression correlated positively with higher tumour grade ($P = 0.0014$, PAb 1801; $P = 0.0004$, p53-BP-12; $P = 0.0012$, DO7; $P = 0.0249$, CM1) (Figure 3). Staining using DO7 correlated significantly with negative ER status, as determined with the anti-ER monoclonal antibody ERLH1 (Henry *et al.*, 1993); in general p53-negative breast carcinomas were more fre-

**Table I** Antibodies specific to p53 protein

| *Antibody* | *Soource* | *Species* | *Dilution* | *Incubation* |
|---|---|---|---|---|
| DO7[b] | Novocastra | M | 1:100 | [a]16 h, 4°C |
| PAb1801[c] | Novocastra | M | 1:40 | [a]16 h, 4°C |
| p53-BP-12[d] | Novocastra | M | 1:50 | 1 h, 25°C |
| CM1[e] | Novocastra | P | 1:200 | 16 h, 4°C |

[a]Microwave antigen retrieval. M, mouse monoclonal antibody; P, rabbit polyclonal antiserum. [b]Bártek *et al.* (1993). [c]Banks *et al.* (1986). [d]Bártek *et al.* (1991). [e]Vojtesek *et al.* (1992).

**Table II** p53 protein immunoreactivity in routinely processed breast carcinoma

| *Antibody* | *No. of positive cases* | *Percentage* |
|---|---|---|
| PAb 1801 | 111/238 | 46.6 |
| p53-BP-12 | 100/232 | 43.0 |
| DO7 | 98/245 | 40.0 |
| CM1 | 91/242 | 37.6 |

**Figure 1** Invasive ductal carcinoma showing intense nuclear immunopositivity for p53 using DO7 (**a**) and PAb 1801 (**b**). Weaker p53 immunostaining is observed with p53-BP-12 (**c**). Note cytoplasmic and background staining using CM1 (**d**).

quently ER positive ($P = 0.028$) (Figure 4). Subsequent examination of the relationship between response to endocrine therapy on relapse and p53 status indicated that positive nuclear immunostaining was correlated inversely with response (chi-square values with Yates' correction: PAb 1801, $\chi^2 = 4.129$; p53-BP-12, $\chi^2 = 4.178$, DO7, $\chi^2 = 5.727$; CM1, $\chi^2 = 4.129$) (Figure 5). Thus in each instance this relationship proved statistically significant (PAb 1801 $P = 0.00064$; p53-BP-12 $P = 0.00069$; DO7 $P = 0.00516$; CM1 $P = 0.00064$). Although the number of patients within this group was small, incorporating only 17 patients, it was notable that no p53-positive individual showed a response to endocrine therapy.

Using the antibody PAb 1801, our results also suggested a relationship between tumour p53 immunostaining and c-jun ($P = 0.0307$). Our data indicated that although most tumours appeared to express c-jun, approximately two-thirds of this group were found to be p53 negative, while in contrast three-quarters of the c-jun-negative tumours were found to be p53 positive. As a result, p53-negative tumours were found to be approximately five times more likely to be c-jun positive than p53-positive tumours. Finally, no significant relationship was observed between p53 expression and c-erbB-2 oncoprotein, retinoblastoma gene protein, EGFR and progesterone receptor expression.

### Discussion

Abnormalities in the p53 gene, either in the form of gene loss or in the form of mutations, are the most common genetic abnormality in primary breast carcinoma (Varley *et al.*, 1991). The human p53 gene has been mapped on the short arm of chromosome 17, where other genes with prognostic significance in breast cancer, such as c-*erb*B-2 and the anti-metastatic gene nm23, are also located (Sawan *et al.*, 1994).

Our study compared one polyclonal and three monoclonal antibodies, which have often been used previously, in isolation, in immunohistochemical studies that have sought to define tumour p53 status. We sought to gain a consensus by applying each of these antibodies in parallel to study p53 expression/stabilisation within a large series of breast carcinomas. The three monoclonal antibodies employed in this comparison, namely DO7, PAb 1801 and p53-BP-12, are each known to react with the amino-terminal portion of the p53 molecule, which is distal to the central region, which is most frequently affected by point mutations. Thus, we would expect a high degree of structual conservation within the N-terminal region reactive with these antibodies. Although each of the monoclonal antibodies recognises the immunodominant N-terminal portion of the p53 molecule, the precise location of their complementary epitopes varies between two sites. Antibody PAb 1801 is known to recognise an epitope mapping between amino acid residues 32 and 79 (Banks *et al.*, 1986) while antibodies BP-53-12 and DO7 react with the same epitope within the region defined by amino acids 20–25 (Stephen *et al.*, 1995). In addition, this N-terminal sequence of amino acids is also known to overlap with a seven amino acid sequence (18–23), that constitutes the binding site for the p53 modulatory protein MDM2 (Picksley *et al.*, 1994).

In the present study, the detection rate of p53 protein in breast carcinoma varied from 37.6% to 46.6%, depending on the anti-p53 antibody used. These results are in keeping with the findings of previous similar studies (Cattoretti *et al.*, 1988; Walker *et al.*, 1991; Barbareshi *et al.*, 1992; Poller *et al.*, 1992; Sawan *et al.*, 1992; Yamashita *et al.*, 1993). However, in other studies the rate of p53-positive breast carcinomas was found to be lower (13–23%) (Iwaya *et al.*, 1991; Spandidos *et al.*, 1992; Trudel *et al.*, 1992; Martinazzi

**Figure 2** Patient survival related to tumour p53 protein expression determined by immunohistochemistry using the monoclonal antibody PAb 1801. (**a**) All patients. (**b**) lymph node-negative subgroup. (**c**) lymph node-positive subgroup.

**Figure 3** p53 protein expression related to breast carcinoma grade (immunostaining with monoclonal antibody PAb 1801). ■, p53 negative; ▨, p53 positive.

*et al.*, 1993). This discrepancy could reflect differences in the fixation of the tissues used and it could be related to the different antibodies used for each study, the sensitivity of the immunohistochemical technique performed and the methods of interpretation of the immunostaining. In our series, the rate of p53 positivity in invasive lobular carcinomas (8.3–27.3%) and in low-grade special types of breast carcinoma was lower than that observed in invasive ductal carcinomas (40.6–49.7%), confirming the results of previous reports (Poller *et al.*, 1992; Martinazzi *et al.*, 1993).

This study has firstly confirmed the prognostic significance of expression of p53 protein in breast cancer but highlights the paramount importance of selection of the most appropriate antibody for immunohistochemistry. We have demonstrated that among the four anti-p53 antibodies used

**Figure 4** p53 protein expression related to tumour oestrogen receptor (ER) immunohistochemical status (monoclonal antibody DO7). ■, p53 negative; ▨, p53 positive.

**Figure 5** Illustration of the relationship observed between tumour p53 immunostaining and response, to endocrine therapy. ■, Positive response to endocrine therapy; ▨, negative response to endocrine therapy.

**Figure 6** Tumour p53 immunostaining related to c-*jun* oncoprotein expression. ■, p53 negative; ▨, p53 positive.

(PAb1801, DO7, p53-BP-12 and CM1), PAb 1801 and to a lesser extent DO7 proved to be the most effective antibodies for the immunodetection of p53 protein in routinely processed material, confirming the results of similar studies in colonic and cervical carcinomas (Baas *et al.*, 1994; Lambkin *et al.*, 1994). Diminished performance of p53-BP-12 relative to 1801 and DO7 could reflect the use of this antibody without associated antigen retrieval. Although preliminary experiments, which optimised the staining conditions for each antibody, indicated that microwave pretreatment of sections enhanced the background cytoplasmic staining produced by p53-BP-12. This effect was not apparent with PAb 1801 or DO7. We have further demonstrated that in this particular series of tumours the p53 immunohistochemical status, as detected by PAb 1801, gives the most significant information with respect to the survival of all patients and of the node-negative subgroup, which presents the greatest dilemma for design of treatment strategies. This finding supports the observation made by Allred *et al.* (1993) in their study of p53 expression in frozen sections of breast tumour tissue from node-negative patients. The degree of separation of the survival curves in our study group, however, is not as great as had been observed by previous workers (Elledge *et al.*, 1993); and in our previous smaller studies using frozen section (Ostrowski *et al.*, 1991) and paraffin section immunohistochemistry (Sawan *et al.*, 1992); the reasons for this are not clear.

There are a number of different possible explanations for differing performances between antibodies in terms of predictive power. One possibility is the sensitivity of the detection system, it is notable that the monoclonal antibody detecting the greatest number of cases positive for p53 exhibits the greatest prognostic power. In general, good correspondence, was observed between the results obtained with DO7 and p53-BP-12 but additional positive cases were recorded using PAb 1801. Whether this difference relates directly to the different epitopes recognised by DO7/p53-BP-12 and PAb 1801 or is a function of antibody avidity and minimum thresholds of detection or even a reflection of reactivity of our detector with different p53 antibody subclasses, cannot be categorically defined from this work. It is also possible that the differences between the antibodies reflect the suggestion that p53 may be present in different tumours in different molecular forms and evidence for this is presented by Spandau (1994), who showed differing patterns of wild-type p53 in the normal epidermis, depending upon the antibody used for immunostaining. Furthermore, differing antibody detection rates could be related to masking of epitopes as a result of p53 binding to other proteins, although it is likely that fixation together with microwave antigen retrieval would eliminate this effect. However, it is interesting to note that each of the monoclonal antibodies reactive with amino acid sequence 20–25 detected fewer positive tumours than the antibody of choice PAb 1801. It is tempting to speculate that this region is more subject to alteration during fixation or complex formation, which may interfere with subsequent reaction of the antibody with its complementary ligand. The polyclonal antibody CM1 proved the least effective antibody in immunolocalising p53. In multiple instances cytoplasmic staining was evident with CM1 in the complete absence of nuclear staining, although each of the monoclonal antibodies produced defined nuclear staining on sections from the same tumour. Equally, a number of tumours scored negative by p53 in immunohistochemistry have been found to express significant levels of p53, which may be detected using quantitative non-competitive ELISAs that employed CM1 as the detector (Thomas *et al.*, 1995). This indicates that CM1 may exhibit reduced sensitivity for p53, and may be more subject to fixation artifacts in immunohistochemistry than any of the three monoclonal antibodies studied. Furthermore, this work has reinforced our findings in respect of differential efficiency of p53 detection using antibodies PAb 1801, DO7 and CM1 to detect p53 in frozen, unfixed tissue sections in direct comparison with p53 levels assessed by ELISA in cytosols prepared from the same tissue.

It has been previously shown that the presence of detectable p53 protein in breast cancer is significantly correlated with poor tumour differentiation and poor patient prognosis (Iwaya *et al.*, 1991; Sawan *et al.*, 1992; Spandidos *et al.*, 1992; Yamashita *et al.*, 1993). It might be proposed that the association of p53 immunostaining with prognosis would reflect its correlation with tumour grade. In this study immunostaining for any of the four antibodies correlated strongly with grade but only PAb 1801 shows significant survival effects. This suggests an effect independent of grade, but we could detect no prognostic effects for p53 in any group stratified for grade, using any antibody.

Previous studies have shown that the presence of detectable p53 protein in breast cancer is significantly correlated with increased levels of c-erbB-2 oncoprotein (Iwaya *et al.*, 1991; Barbareschi *et al.*, 1992; Poller *et al.*, 1992; Spandidos *et al.*, 1992; Martinazzi *et al.*, 1993), and EGFR (Walker *et al.*, 1991; Poller *et al.*, 1992), but similar correlations were not observed in our material. Correlation between Rb and p53 structural abnormalities has been reported in breast carcinoma and in several other malignant tumours (Hall and Lane, 1994). In our study and in others, no correlation was found between the expression of Rb and p53 genes at the cellular level. (Walker *et al.*, 1991; Trudel *et al.*, 1992). However, within this same series of tumours a significant relationship was observed between expression of Rb and cyclin D1 (McIntosh *et al.*, 1995). An interesting finding reported in the current study for the first time, was the significant correlation of the immunolocalisation of p53 protein with the apparent absence of c-jun oncoprotein in the breast cancer cells.

Of great interest is our observation that p53 overexpression (as determined by any of the four antibodies), is associated with patients' failure to respond to endocrine therapy. While it is true that p53-positive tumours tend to be negative for ER and to be of high grade, this observation clearly requires further investigation since our numbers are small. Currently cases assembled from the Northern Region hospitals are being examined in order to enable us to more fully test this relation. The possibility that p53 immunostaining can provide objective supplementary predictive information for endocrine response is an attractive one.

A further contentious issue regarding p53 protein overexpression concerns the relationship to p53 mutation. It is now well established that although mutation and overexpression are correlated (Baas *et al.*, 1994), a high degree of discordance is observed (Jacquemier *et al.*, 1994; Xu *et al.*, 1994).

Immunodetection of p53 protein has been recently reported in normal cells following genotoxic injury, which leads to stabilisation of the wild-type p53 (MacKay *et al.*, 1988). The evaluation of the results of the immunohistochemical detection of p53 protein, should be carried out with great care since the observed tumour immunophenotypes do not always reflect the underlying biochemical and biological cellular changes. It will clearly be of importance to compare the prognostic significance of mutation *per se* to overexpression determined by immunostaining and this is now proceeding in our laboratory. In addition, since we have demonstrated the differing prognostic significance of p53 determined by various antibodies, it will now be important to investigate the significance of the relationship between such staining differences (perhaps reflecting different molecular forms), specific mutations and survival.

It is clear that in the study of p53 alterations in human tumours, it is no longer sufficient to designate cases as 'p53 positive'. Rather, the molecular change must be carefully defined by the antibody used for immunohistochemistry or by DNA analysis for mutation, and ideally both. In addition, assessment of expression of DNA binding proteins such as MDM2 may be important.

This study demonstrates that the relationship of p53 over expression to clinical outcome is complex, and that more comprehensive analysis is required to translate the results into reliable clinical guidelines.

### References

ALLDRED DC, CLARK GM, ELLEDGE R, FUQUA SAW, BROWN RW, CHAMNESS GC, OSBORNE CK AND MCGUIRE WL. (1993). Association of p53 protein expression with tumour cell proliferation rate and clinical outcome in node negative breast cancer. *J. Natl Cancer Inst.*, **85**, 200-206.

ANDERSON JJ, TINIAKOS D, HORNE G, MELLON K, ANGUS B AND HORNE CHW. (1994). Production and characterization of retinoblastoma gene product specific monoclonal antibodies for use on formalin-fixed, paraffin-embedded tissues. *J. Pathol.*, **172** suppl, 135A.

BAAS IO, MULDER J-W, OFFERHAUS GJA, VOGELSTEIN B AND HAMILTON SR. (1994). An evaluation of six antibodies for immunohistochemistry of mutant p53 gene product in archival colorectal neoplasms. *J. Pathol.* **172**, 5-12.

BAKER SJ, FEARON ER, NIGRO JM, HAMILTON SR, PREISINGER AC, JESSUP JM, VANTUINEN P, LEDBETTER DH, BARKER DF, NAKAMURA Y, WHITE R AND VOGELSTEIN B. (1989). Chromosome 17 deletions and p53 gene mutations in colorectal carcinomas. *Science*, **244**, 217-221.

BANKS L, MATLASHEWSKI G AND CRAWFORD L. (1986). Isolation of human p53 specific monoclonal antibodies and their use in the study of human p53 expression. *Eur. J. Biochem.*, **159**, 529-534.

BARBARESCHI M, LEONARDI E, MAURI FA, SERIO G AND PALMA PD. (1992). p53 and c-*erb*B-2 expression in breast carcinomas. An immunohistochemical study including correlations with receptor status, proliferation markers, and clinical stage in human breast cancer. *Am. J. Clin. Pathol.*, **98**, 408-418.

BARNES DM. (1989). Breast cancer and a proto-oncogene. c-*erb*B-2 is a reliable prognostic marker. *Br. Med. J.*, **299**, 1061.

BARNES DM, DUBLIN EA, FISHER CJ, LEVISON DA AND MILLIS RR. (1993). Immunohistochemical detection of p53 protein in mammary carcinoma. *Hum. Pathol.*, **24**, 469-476.

BÁRTEK J, BÁRTKOVÁ J, VOIJESEK B, STASKOVA Z, LUKAS J, REJTHAR A, KOVARIK J, MIDGLEY CA, GANNON JV AND LANE DP. (1991). Aberrant expression of the p53 onco-protein is a common feature of a wide spectrum of human malignancies. *Oncogene*, **6**, 1699-1703.

BARTEK J, BÁRTKOVÁ J, LUKÁS J, STASKOVÁ Z, VOJTESEK B AND LANE DP. (1993). Immunohistochemical analysis of the p53 oncoprotein on paraffin sections using a series of novel monoclonal antibodies. *J. Pathol.*, **169**, 27-34.

CATTORETTI G, RILKE F, ANDEROLA S, DAMATO L AND DELIA D. (1988). p53 expression in breast cancer. *Int. J. Cancer*, **41**, 178-183.

CORBETT IP, HENRY JA, ANGUS B, WATCHORN CJ, WILKINSON L, HENNESSEY C, GULICK WJ, TUZI NL, MAY FEB, WESTLEY BR AND HORNE CHW. (1990). NCL-CB11, a new monoclonal antibody recognising the internal domain of the c-*erb*B-2 oncogene protein effective for use on formalin-fixed, paraffin-embedded tissues. *J. Pathol.*, **161**, 15-25.

EARLY BREAST CANCER TRIALISTS' COLLABORATIVE GROUP. (1992). Systemic treatment of early breast cancer by hormonal, cytotoxic, or immune therapy. *Lancet*, **339**, 1-15, 71-85.

ELLEDGE RM, FUQUA SAW, CLARK GM, PUJOL P, ALLRED G AND MCGUIRE WL. (1993). Prognostic significance of p53 gene alterations in node-negative breast cancer. *Breast Cancer Res. Treat.*, **26**, 225-235.

ELSTON CW AND ELLIS IO. (1991). The Nottingham Prognostic Index in primary breast cancer. *Breast Cancer Res. Treat.*, **22**, 207-219.

HALL PA AND LANE DP. (1994). p53 in tumour pathology; can we trust immunohistochemistry? Revisited! *J. Pathol.*, **172**, 1-4.

HENRY JA, PIGGOTT NH, MALLICK UK, NICHOLSON S, FARNDON JJ, WESTLEY BR AND MAY FEB. (1991). pNR-2/pS2 immunohistochemical staining in breast cancer: correlation with prognostic factors and endocrine response. *Br. J. Cancer*, **63**, 615-622.

HENRY L, ANGUS B, LENNARD TWJ. (1993). New monocloncal antibodies to oestrogen and progesterone receptor: characterization and clinical studies in human breast cancer. *J. Pathol.*, **169** suppl, 168A.

IWAYA K, TSUDA H, HIRAIDE H, TAMAKI K, TAKAMURA S, FUKUTOMI T, MUKAI K AND HIROHASHI S. (1991). Nuclear p53 immunoreaction associated with poor prognosis of breast ccancer. *Jpn. J. Cancer Res.*, **82**, 835-840.

JACQUIEMER J, MOLÈS JP, PENAULT-LLORCA F, ADÉLAIDE J, TORRENTE M, VIENS P, BIRNBAUM D AND THEILLET C. (1994). p53 immunohistochemical analysis in breast cancer with four monoclonal antibodies: comparison of staining and PCR-SSCP results. *Br. J. Cancer*, **69**, 846-852.

KERN SE, PIETENPOL JA, THAINGALINGHAM S, SEYMOUR A, KINXLER KW AND VOGELSTEIN B. (1992). Oncogenic form of p53 inhibit p53-regulated gene expression. *Science*, **256**, 827-830.

LANE DP. (1992). p53, guardian of the genome. *Nature*, **358**, 15-16.

LANE DP AND BENCHIMAL S. (1990). p53 oncogene or anti-oncogene? *Gene Dev.*, **4**, 1-8.

LAMBKIN HA, MOTHERSILL CM AND KELEHAN P.(1994). Variations in immunohistochemical detection of p53 protein overexpression in cervical carcinomas with different antibodies and methods of detection. *J. Pathol.*, **172**, 13-18.

LEVINE AJ, MOMAND J AND FINLAY CA. (1991). The p53 tumour-suppressor gene. *Nature*, **351**, 453-456.

MCINTOSH GG, ANDERSON JJ, MILTON I, STEWARD M, PARR AH, THOMAS MD, HENRY JA, ANGUS B AND HORNE CHW. (1995). Determination of the prognostic value of cyclin D1 overexpression in breast cancer. *Oncogene*, **11**, 885-891.

MACKAY J, STEEL CM, ELDER DA, FOREST APM AND EVANS HJ. (1988). Allele loss on short arm of chromosome 17 in breast cancers. *Lancet*, **2**, 1384-1385.

MARTINAZZI M, CRIVELLI F, ZAMPATTI C AND MARTINAZZI S. (1993). Relationship between p53 expression and other prognostic factors in human breast carcinoma. An immunohistochemical study. *Am. J. Clin. Pathol.*, **100**, 213-217.

MULLIGAN LM, MATLASHEWSKI GJ, SCRABLE HJ AND CAVENEE WK. (1990). Mechanisms of p53 loss on human sarcomas. *Proc. Natl Acad. Sci. USA*, **87**, 5863-5867.

NEVILLE AM. (1990). Are breast cancer axillary node mcirometastases worth detecting (editorial). *J. Pathol.*, **161**, 283-284.

OSTROWSKI JL, SAWAN A, HENRY L, WRIGHT C, HENNESSEY RA, LENNARD TJW, ANGUS B AND HORNE CHW. (1991). p53 expression in human breast cancer related to survival and prognostic factors: an immunohistochemical study. *J. Pathol.*, **164**, 75-81.

PICKSLEY SM, VOJTESEK B, SPARKS A AND LANE D. (1994). Immunochemical analysis of the interaction of p53 with mdm2; Fine mapping of the mdm2 binding site on p53 using synthetic peptides. *Oncogene*, **9**, 2523-2529.

POLLER DN, HUTCHINGS CE, GALEA M, BEEL JA, NICHOLSON RA, ELSTON CW, BLAMEY RW AND ELLIS IO. (1992). p53 protein expression in human breast carcinoma: relationship to expression of epidermal growth factor receptor, c-*erb*B-2 protein overexpression, and oestrogen receptor. *Br. J. Cancer*, **66**, 583-588.

PIGGOTT NH, HENRY JA, MAY FEB AND WESTLEY BR. (1991). Anti-peptide antibodies against the pNR-2 oestrogen-regulated protein of human breast cancer cells and detection of pNR-2 expression in normal tissues by immunohistochemistry. *J. Pathol.*, **163,** 95-104.

SAWAN A, RANDALL B, ANGUS B, WRIGHT C, HENRY JA, OSTROWSKI J, HENNESSEY C, LENNARD TJW, CORBETT I AND HORNE CHW. (1992). Retinoblastoma and p53 gene expression related to relapse and survival in human breast cancer: an immunohistochemical study. *J. Pathol.*, **168,** 23-28.

SAWAN A, LASCU I, VERON M, ANDERSON JJ, WRIGHT C, HORNE CHW AND ANGUS B. (1994). nm23/NDPK expression human breast cancer in relation to relapse, survival and other prognostic factors: an immunohistochemical study. *J. Pathol.*, **172,** 27-34.

SHI SR, KEY ME AND KARLA KL. (1991). Antigen retrieval in formalin-fixed, paraffin-embedded tissues: an enhancement method for immunohistochemical staining based on microwave oven heating of tissue sections. *J. Histochem. Cytochem.*, **39,** 741-748.

SPANDAU DF. (1994). Distinct conformations of p53 are observed at different stages of keratinocyte differentiation. *Oncogene*, **9,** 1861-1868.

SPANDIDOS DA, KARIOSSIFIDI H, MALLIRI A, LINDARDOPOULOS S, VASSILARIOS S, TSIKKINIS A AND FEILD JK. (1992). Expression of *ras*, Rb1 and p53 proteins in human breast cancer. *Anticancer Res.*, **12,** 81-90.

STEPHEN CW, HEIMINEN P AND LANE DP. (1995). Characterization of epitopes on human p53 using phage displayed peptide libraries: Insights into antibody peptide interactions. *J. Mol. Biol.*, **248,** 58-78.

THOMAS MD, PARR A, MCINTOSH GG, ANDERSON JJ, ELLIS I, NICHOLSON R, HORNE CHW AND ANGUS BA. (1995). Measurement of p53 protein in human breast cancer: comparison between immunohistochemistry and semi-quantitative ELISA values. *J. Pathol.*, **176** suppl, 172.

TINIAKOS D, SCOTT L, CORBETT IP, PIGGOTT NH AND HORNE CHW. (1994). Studies of c-jun oncogene expression in human breast using a new monoclonal antibody, NCL-DK4. *J. Pathol.*, **172,** 19-26.

TRUDEL M, MULLIGAN L, CAVERNEE W, MARGOLESE R, COTE J AND GARIEPY G. (1992). Retinoblastoma and p53 gene product expression in breast carcinoma. *Hum. Pathol.*, **23,** 1388-1394.

VARLEY JM, BRAMMAR WJ, LANE DP, SWALLOW JE, DOLAN C AND WALKER RA. (1991). Loss of chromosome 17p13 sequences and mutation of p53 in human breast carcinomas. *Oncogene*, **6,** 413-421.

VOGELSTEIN B AND KINZLER KW. (1992). p53 function and dysfunction. *Cell*, **70,** 523-526.

VOJTESEK B, BÁRTEK J, MIDGLEY CA AND LANE DP. (1992). An immunochemical analysis of the human-nuclear phosphoprotein p53. New monoclonal antibodies and epitope mapping using recombinant p53. *J. Immunol. Methods*, **151,** 237-244.

WALKER RA, DEARING SJ, LANE DP AND VARLEY JM. (1991). Expression of p53 protein in infiltrating and *in-situ* breast carcinomas. *J. pathol.*, **165,** 203-211.

XU L, CHEN Y-T, HUVOS AG, ZLOTOLOW IM, RETTIG WJ, OLD LJ AND GARIN-CHESA P. (1994). Overexpression of p53 protein in squamous cell carcinomas of head and neck without apparent gene mutations. *Diagm. Mol. Pathol.*, **3,** 83-92.

YAMASHITA H, KOBAYASHI S, IWASE E, ITOH Y, KUZUSHIMA T, IWATA H, ITOH K, HAITO A, YAMASHITA T, MASAOKA A AND-KIMURA N. (1993). Analysis of oncogenes and tumour suppressor genes in human breast cancer. *Jpn. J. Cancer Res.*, **84,** 871-878.


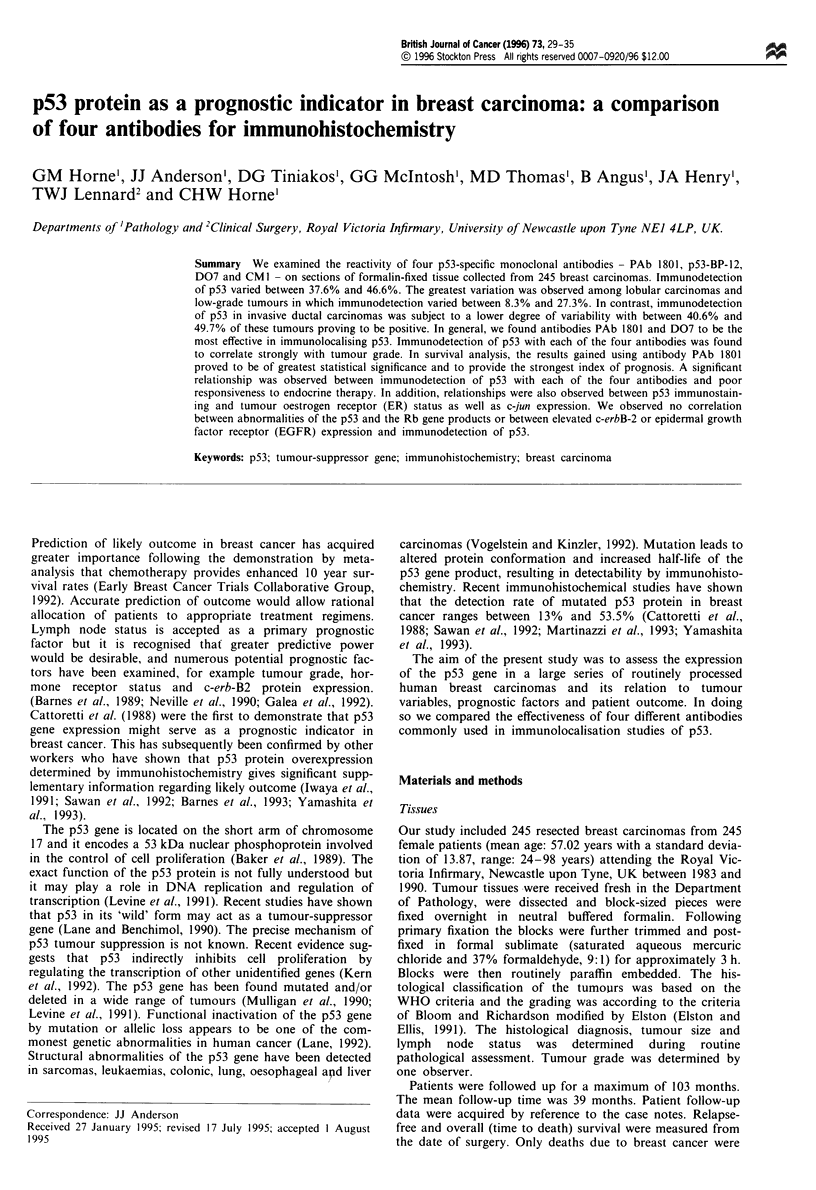

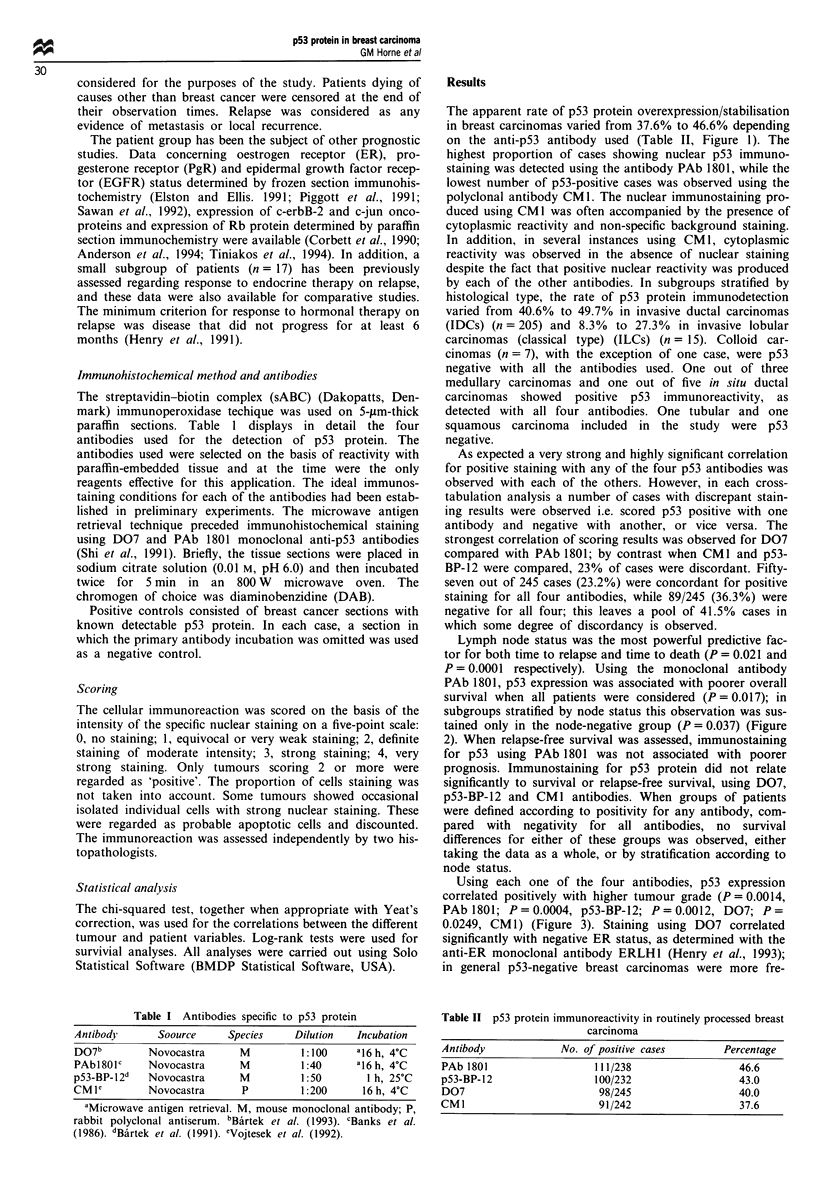

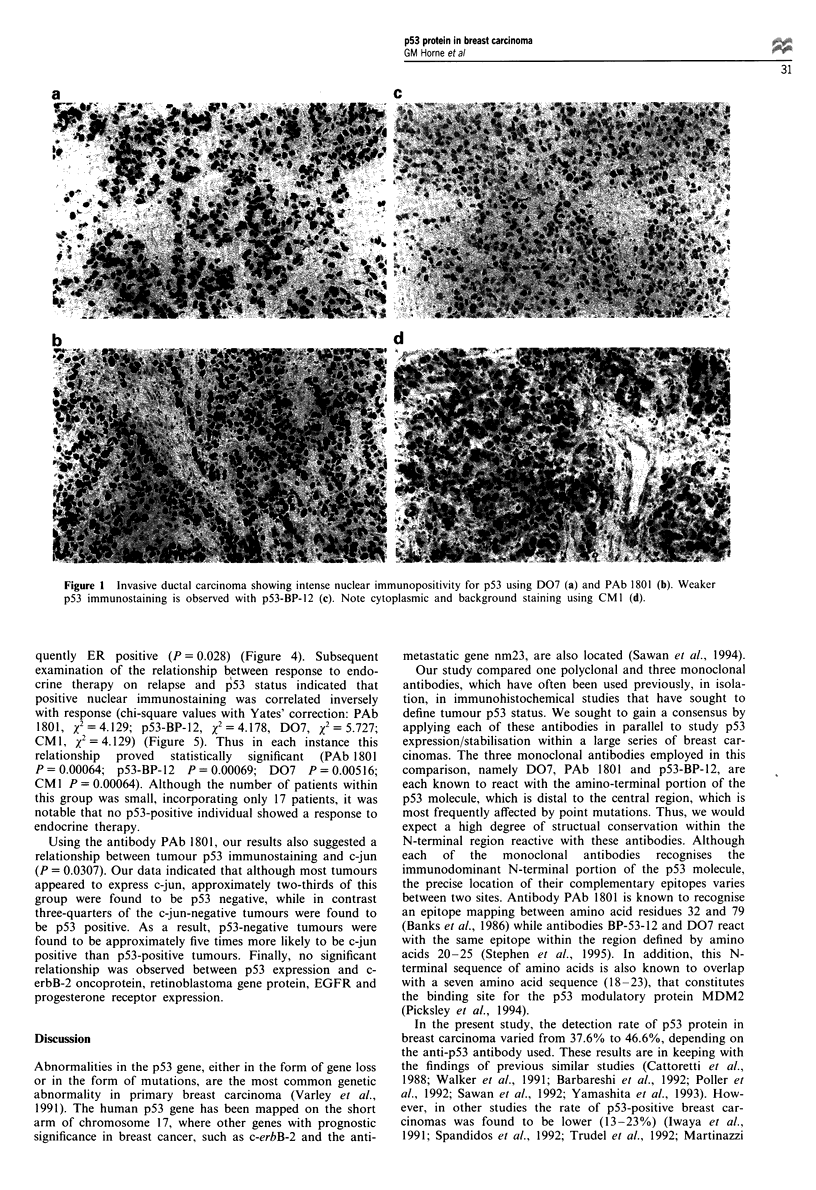

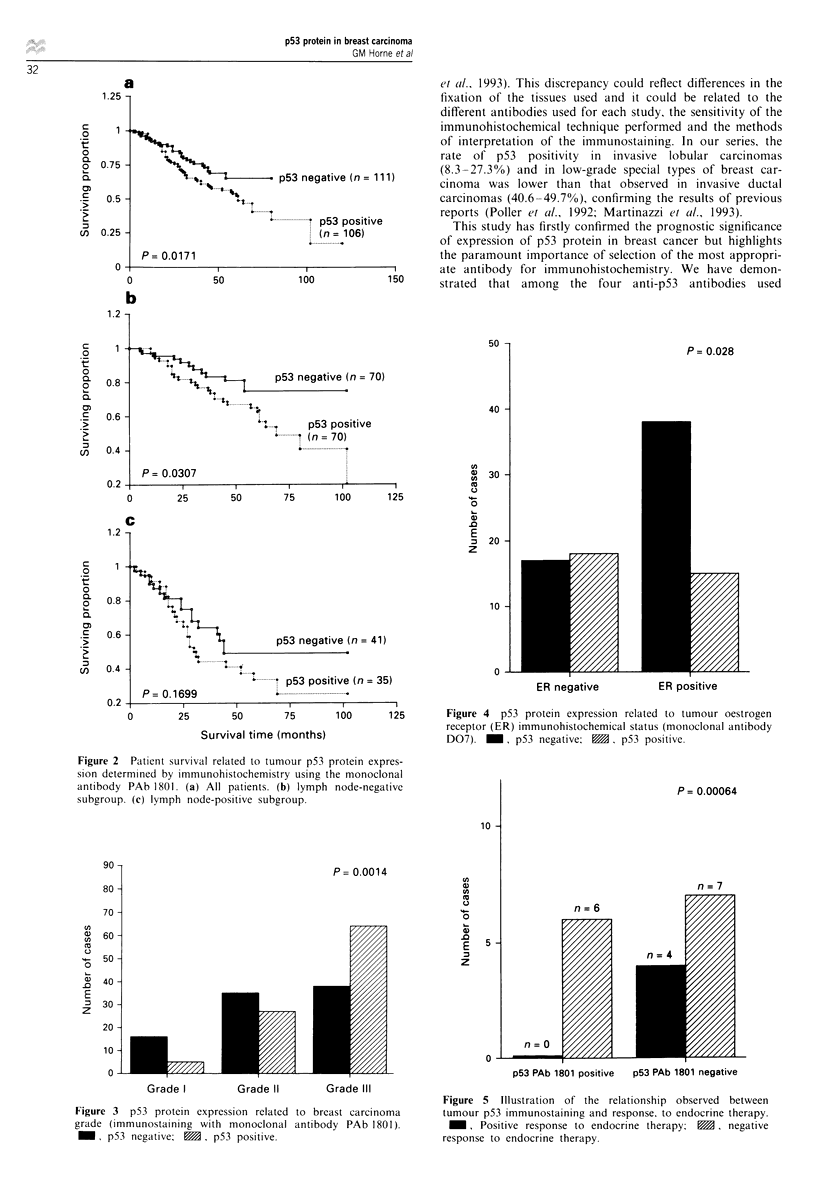

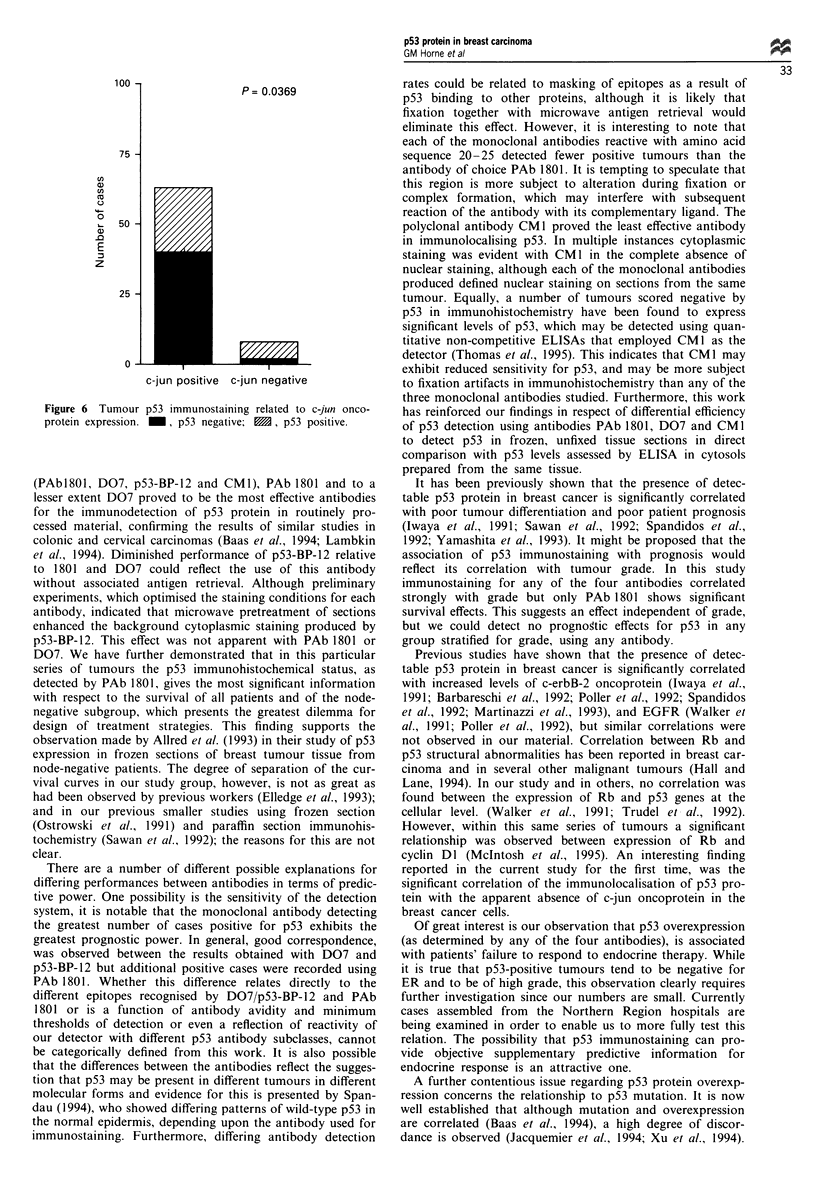

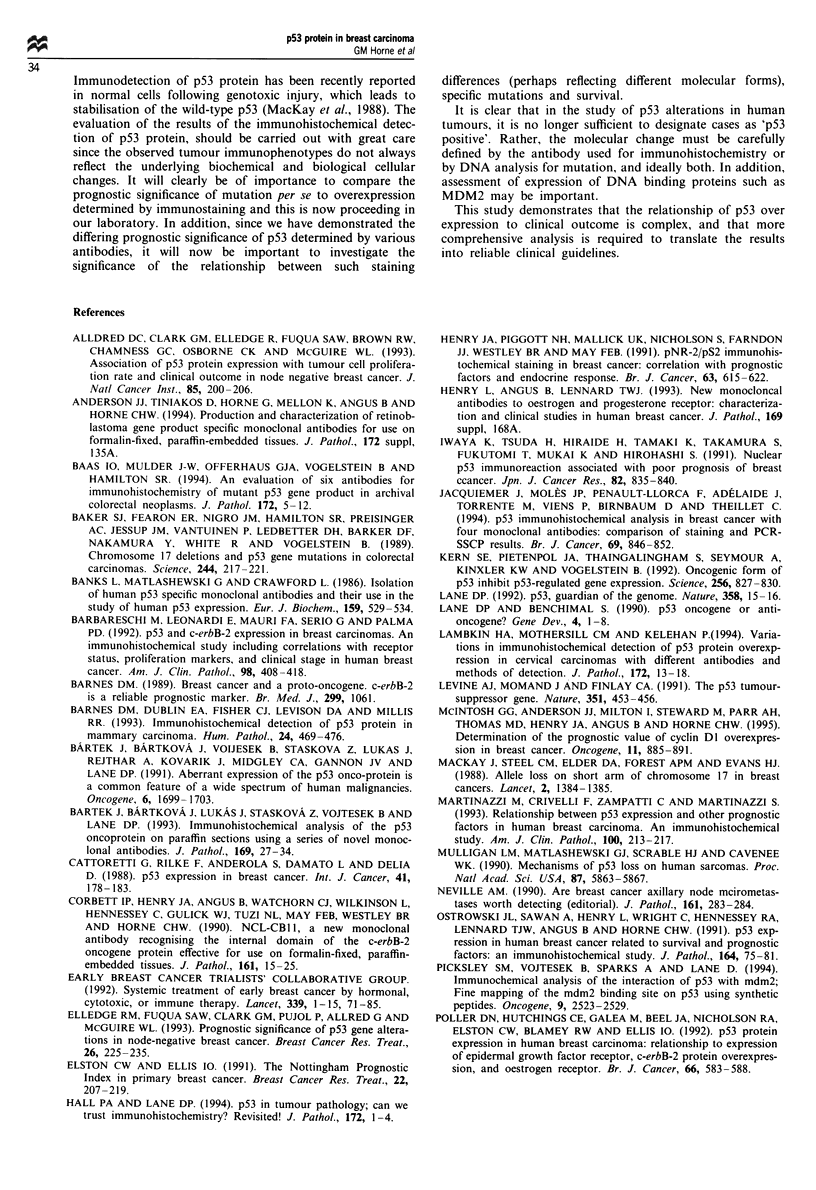

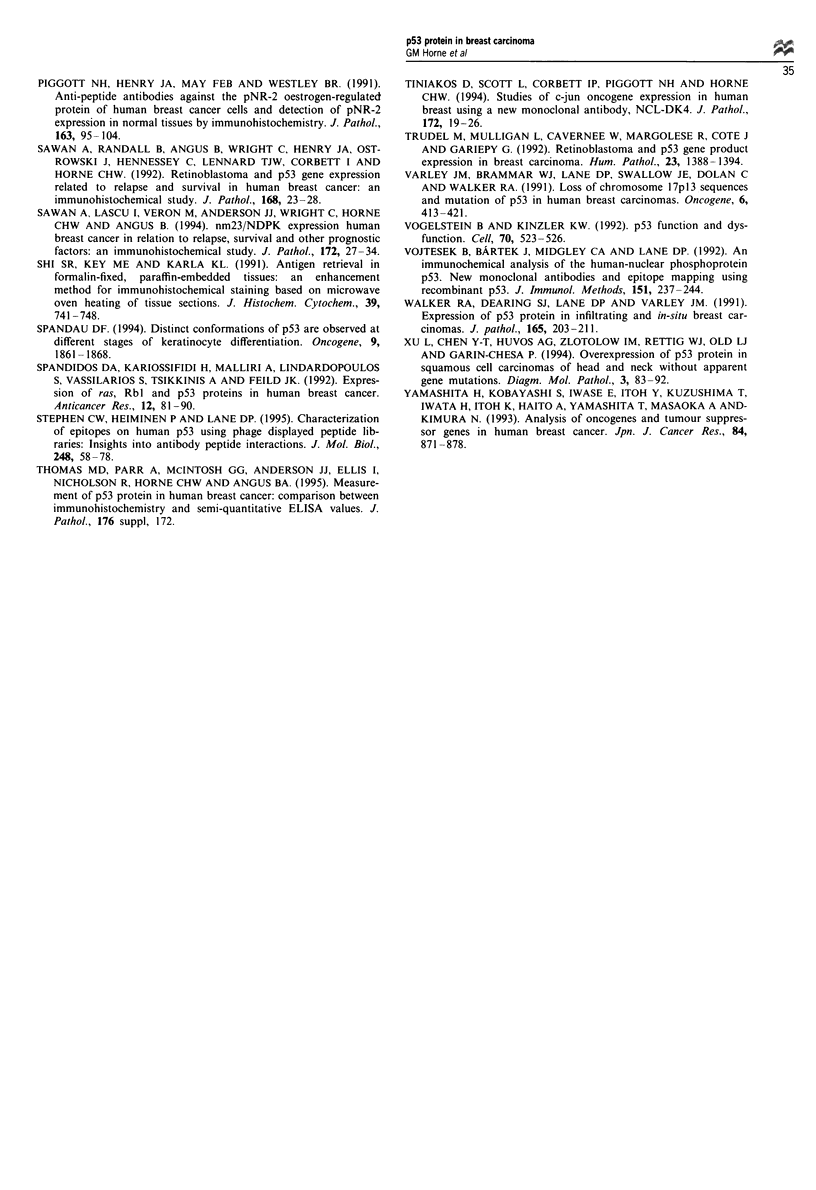


## References

[OCR_00857] Allred D. C., Clark G. M., Elledge R., Fuqua S. A., Brown R. W., Chamness G. C., Osborne C. K., McGuire W. L. (1993). Association of p53 protein expression with tumor cell proliferation rate and clinical outcome in node-negative breast cancer.. J Natl Cancer Inst.

[OCR_00873] Baas I. O., Mulder J. W., Offerhaus G. J., Vogelstein B., Hamilton S. R. (1994). An evaluation of six antibodies for immunohistochemistry of mutant p53 gene product in archival colorectal neoplasms.. J Pathol.

[OCR_00879] Baker S. J., Fearon E. R., Nigro J. M., Hamilton S. R., Preisinger A. C., Jessup J. M., vanTuinen P., Ledbetter D. H., Barker D. F., Nakamura Y. (1989). Chromosome 17 deletions and p53 gene mutations in colorectal carcinomas.. Science.

[OCR_00886] Banks L., Matlashewski G., Crawford L. (1986). Isolation of human-p53-specific monoclonal antibodies and their use in the studies of human p53 expression.. Eur J Biochem.

[OCR_00888] Barbareschi M., Leonardi E., Mauri F. A., Serio G., Dalla Palma P. (1992). p53 and c-erbB-2 protein expression in breast carcinomas. An immunohistochemical study including correlations with receptor status, proliferation markers, and clinical stage in human breast cancer.. Am J Clin Pathol.

[OCR_00895] Barnes D. M. (1989). Breast cancer and a proto-oncogene.. BMJ.

[OCR_00901] Barnes D. M., Dublin E. A., Fisher C. J., Levison D. A., Millis R. R. (1993). Immunohistochemical detection of p53 protein in mammary carcinoma: an important new independent indicator of prognosis?. Hum Pathol.

[OCR_00914] Bártek J., Bártková J., Lukás J., Stasková Z., Vojtesek B., Lane D. P. (1993). Immunohistochemical analysis of the p53 oncoprotein on paraffin sections using a series of novel monoclonal antibodies.. J Pathol.

[OCR_00906] Bártek J., Bártková J., Vojtesek B., Stasková Z., Lukás J., Rejthar A., Kovarík J., Midgley C. A., Gannon J. V., Lane D. P. (1991). Aberrant expression of the p53 oncoprotein is a common feature of a wide spectrum of human malignancies.. Oncogene.

[OCR_00919] Cattoretti G., Rilke F., Andreola S., D'Amato L., Delia D. (1988). P53 expression in breast cancer.. Int J Cancer.

[OCR_00925] Corbett I. P., Henry J. A., Angus B., Watchorn C. J., Wilkinson L., Hennessy C., Gullick W. J., Tuzi N. L., May F. E., Westley B. R. (1990). NCL-CB11, a new monoclonal antibody recognizing the internal domain of the c-erbB-2 oncogene protein effective for use on formalin-fixed, paraffin-embedded tissue.. J Pathol.

[OCR_00938] Elledge R. M., Fuqua S. A., Clark G. M., Pujol P., Allred D. C., McGuire W. L. (1993). Prognostic significance of p53 gene alterations in node-negative breast cancer.. Breast Cancer Res Treat.

[OCR_00943] Galea M. H., Blamey R. W., Elston C. E., Ellis I. O. (1992). The Nottingham Prognostic Index in primary breast cancer.. Breast Cancer Res Treat.

[OCR_00952] Henry J. A., Piggott N. H., Mallick U. K., Nicholson S., Farndon J. R., Westley B. R., May F. E. (1991). pNR-2/pS2 immunohistochemical staining in breast cancer: correlation with prognostic factors and endocrine response.. Br J Cancer.

[OCR_00965] Iwaya K., Tsuda H., Hiraide H., Tamaki K., Tamakuma S., Fukutomi T., Mukai K., Hirohashi S. (1991). Nuclear p53 immunoreaction associated with poor prognosis of breast cancer.. Jpn J Cancer Res.

[OCR_00968] Jacquemier J., Molès J. P., Penault-Llorca F., Adélaide J., Torrente M., Viens P., Birnbaum D., Theillet C. (1994). p53 immunohistochemical analysis in breast cancer with four monoclonal antibodies: comparison of staining and PCR-SSCP results.. Br J Cancer.

[OCR_00978] Kern S. E., Pietenpol J. A., Thiagalingam S., Seymour A., Kinzler K. W., Vogelstein B. (1992). Oncogenic forms of p53 inhibit p53-regulated gene expression.. Science.

[OCR_00984] Lambkin H. A., Mothersill C. M., Kelehan P. (1994). Variations in immunohistochemical detection of p53 protein overexpression in cervical carcinomas with different antibodies and methods of detection.. J Pathol.

[OCR_00980] Lane D. P. (1992). Cancer. p53, guardian of the genome.. Nature.

[OCR_00992] Levine A. J., Momand J., Finlay C. A. (1991). The p53 tumour suppressor gene.. Nature.

[OCR_01000] Mackay J., Steel C. M., Elder P. A., Forrest A. P., Evans H. J. (1988). Allele loss on short arm of chromosome 17 in breast cancers.. Lancet.

[OCR_01007] Martinazzi M., Crivelli F., Zampatti C., Martinazzi S. (1993). Relationship between p53 expression and other prognostic factors in human breast carcinoma. An immunohistochemical study.. Am J Clin Pathol.

[OCR_00997] McIntosh G. G., Anderson J. J., Milton I., Steward M., Parr A. H., Thomas M. D., Henry J. A., Angus B., Lennard T. W., Horne C. H. (1995). Determination of the prognostic value of cyclin D1 overexpression in breast cancer.. Oncogene.

[OCR_01011] Mulligan L. M., Matlashewski G. J., Scrable H. J., Cavenee W. K. (1990). Mechanisms of p53 loss in human sarcomas.. Proc Natl Acad Sci U S A.

[OCR_01018] Neville A. M. (1990). Are breast cancer axillary node micrometastases worth detecting?. J Pathol.

[OCR_01022] Ostrowski J. L., Sawan A., Henry L., Wright C., Henry J. A., Hennessy C., Lennard T. J., Angus B., Horne C. H. (1991). p53 expression in human breast cancer related to survival and prognostic factors: an immunohistochemical study.. J Pathol.

[OCR_01025] Picksley S. M., Vojtesek B., Sparks A., Lane D. P. (1994). Immunochemical analysis of the interaction of p53 with MDM2;--fine mapping of the MDM2 binding site on p53 using synthetic peptides.. Oncogene.

[OCR_01041] Piggott N. H., Henry J. A., May F. E., Westley B. R. (1991). Antipeptide antibodies against the pNR-2 oestrogen-regulated protein of human breast cancer cells and detection of pNR-2 expression in normal tissues by immunohistochemistry.. J Pathol.

[OCR_01034] Poller D. N., Hutchings C. E., Galea M., Bell J. A., Nicholson R. A., Elston C. W., Blamey R. W., Ellis I. O. (1992). p53 protein expression in human breast carcinoma: relationship to expression of epidermal growth factor receptor, c-erbB-2 protein overexpression, and oestrogen receptor.. Br J Cancer.

[OCR_01058] Sawan A., Lascu I., Veron M., Anderson J. J., Wright C., Horne C. H., Angus B. (1994). NDP-K/nm23 expression in human breast cancer in relation to relapse, survival, and other prognostic factors: an immunohistochemical study.. J Pathol.

[OCR_01051] Sawan A., Randall B., Angus B., Wright C., Henry J. A., Ostrowski J., Hennessy C., Lennard T. W., Corbett I., Horne C. H. (1992). Retinoblastoma and p53 gene expression related to relapse and survival in human breast cancer: an immunohistochemical study.. J Pathol.

[OCR_01062] Shi S. R., Key M. E., Kalra K. L. (1991). Antigen retrieval in formalin-fixed, paraffin-embedded tissues: an enhancement method for immunohistochemical staining based on microwave oven heating of tissue sections.. J Histochem Cytochem.

[OCR_01069] Spandau D. F. (1994). Distinct conformations of p53 are observed at different stages of keratinocyte differentiation.. Oncogene.

[OCR_01080] Stephen C. W., Helminen P., Lane D. P. (1995). Characterisation of epitopes on human p53 using phage-displayed peptide libraries: insights into antibody-peptide interactions.. J Mol Biol.

[OCR_01093] Tiniakos D. G., Scott L. E., Corbett I. P., Piggott N. H., Horne C. H. (1994). Studies of c-jun oncogene expression in human breast using a new monoclonal antibody, NCL-DK4.. J Pathol.

[OCR_01100] Trudel M., Mulligan L., Cavenee W., Margolese R., Côté J., Gariépy G. (1992). Retinoblastoma and p53 gene product expression in breast carcinoma: immunohistochemical analysis and clinicopathologic correlation.. Hum Pathol.

[OCR_01102] Varley J. M., Brammar W. J., Lane D. P., Swallow J. E., Dolan C., Walker R. A. (1991). Loss of chromosome 17p13 sequences and mutation of p53 in human breast carcinomas.. Oncogene.

[OCR_01110] Vogelstein B., Kinzler K. W. (1992). p53 function and dysfunction.. Cell.

[OCR_01114] Vojtesek B., Bártek J., Midgley C. A., Lane D. P. (1992). An immunochemical analysis of the human nuclear phosphoprotein p53. New monoclonal antibodies and epitope mapping using recombinant p53.. J Immunol Methods.

[OCR_01120] Walker R. A., Dearing S. J., Lane D. P., Varley J. M. (1991). Expression of p53 protein in infiltrating and in-situ breast carcinomas.. J Pathol.

[OCR_01123] Xu L., Chen Y. T., Huvos A. G., Zlotolow I. M., Rettig W. J., Old L. J., Garin-Chesa P. (1994). Overexpression of p53 protein in squamous cell carcinomas of head and neck without apparent gene mutations.. Diagn Mol Pathol.

[OCR_01129] Yamashita H., Kobayashi S., Iwase H., Itoh Y., Kuzushima T., Iwata H., Itoh K., Naito A., Yamashita T., Masaoka A. (1993). Analysis of oncogenes and tumor suppressor genes in human breast cancer.. Jpn J Cancer Res.

